# Supplementary figures and images for: A lipid-free and insulin-supplemented medium supports De Novo fatty acid synthesis gene activation in melanoma cells
Source: PLoS One. 2019 Apr 10;14(4):e0215022. doi: 10.1371/journal.pone.0215022 (PMC6457551; doi:10.1371/journal.pone.0215022)

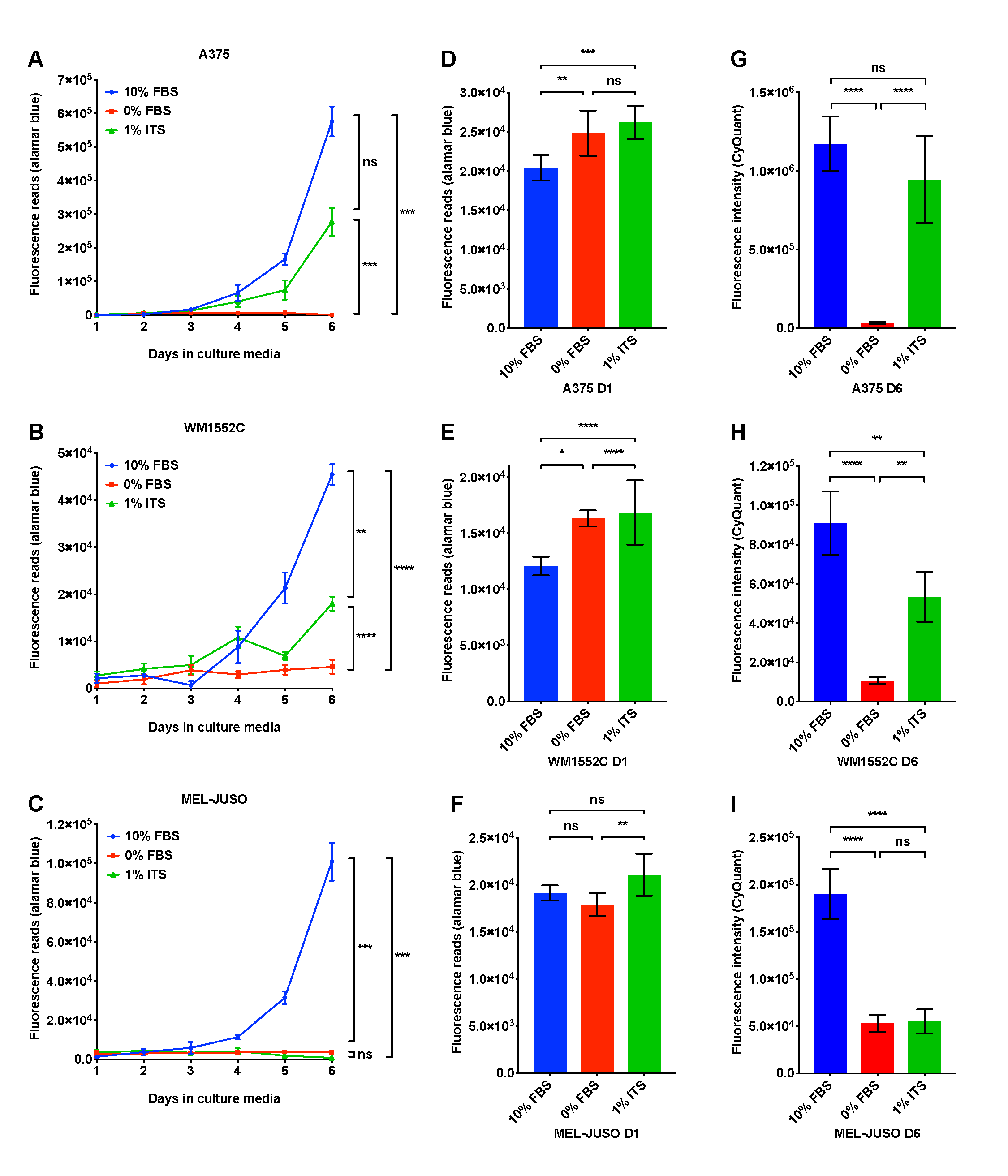

Supplement: S1 Fig — (A–C) A375, WM1552C and MEL-JUSO cell lines were seeded in 10% FBS medium at day zero. On day one, cells were washed with PBS and transferred to the indicated medium conditions. Cell proliferation was measured with alamarBlue assay daily. Each data point represents the mean ±SD of quadruplicate samples. The results were analyzed using two-way repeated measures ANOVA followed by post hoc Tukey’s multiple comparison tests. (A) A375 cells: for culture time, F = 865.8, P < 0.0001; for culture condition, F = 282.8, P < 0.0001; for interaction between culture time and condition, F = 278.7, P < 0.0001. (B) WM1552C cells: for culture time, F = 351.1, P < 0.0001; for culture condition, F = 172.8, P < 0.0001; for interaction between culture time and condition, F = 137.4, P < 0.0001. (C) MEL-JUSO cells: for culture time, F = 501.6, P < 0.0001; for culture condition, F = 475.9, P < 0.0001; for interaction between culture time and condition, F = 584.5, P < 0.0001. (D–F) A375, WM1552C and MEL-JUSO cells were seeded in 10% FBS medium at day zero. On day one, cells were washed with PBS and changed to the indicated medium conditions. alamarBlue assay was performed on the cells cultured in the indicated medium for one hour. Results were analyzed using one-way ANOVA followed by post hoc Tukey’s multiple comparison tests. (D) A375 cells, F = 13.24, P = 0.0003. (E) WM1552C cells, F = 17.31, P < 0.0001. (F) MEL-JUSO cells, F = 6.985, P = 0.0061. Significant differences between medium conditions are indicated as *P < 0.05, **P < 0.01, ***P < 0.001 and ****P < 0.0001. ns, not significant. (G–I) A375, WM1552C and MEL-JUSO cell lines were seeded in 10% FBS medium at day zero. On day one, cells were cultured in the indicated medium conditions. CyQuant assays were performed on the cells cultured in the indicated medium at day six. Each data bar represents average measurement of five replicate samples. Results were analyzed using one-way ANOVA followed by post hoc Tukey’s multiple comparison te [file pone.0215022.s001.tif]

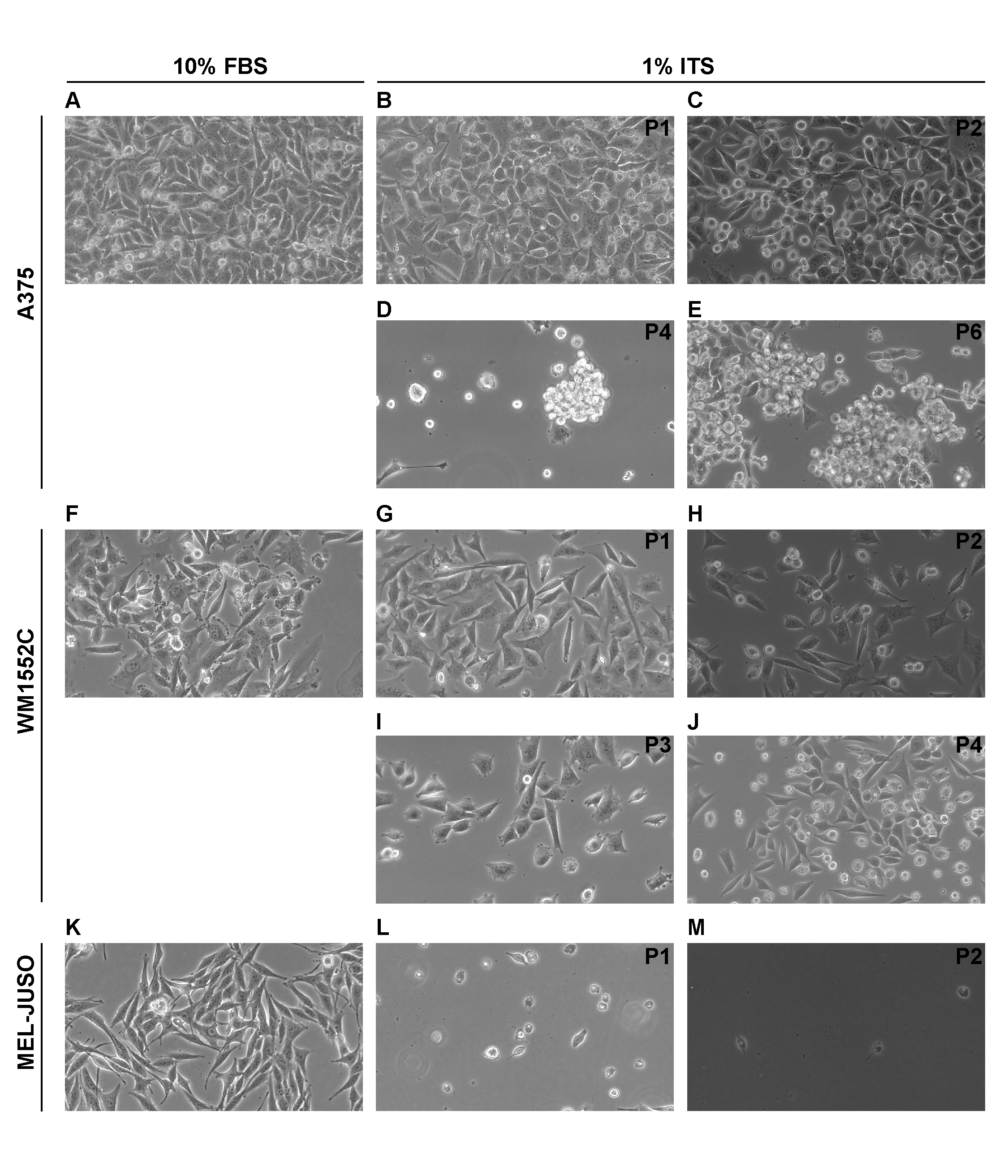

Supplement: S2 Fig — (A, F, K) A375, WM1552C and MEL-JUSO cell lines were routinely maintained in RPMI medium with 10% FBS. Morphologies of cells were recorded by light microscopy. (B-E) Morphologies of A375 cells cultured in 1% ITS medium from passage one (P1) to passage six (P6) over the course of six weeks were monitored by light microscopy. (G-J) Morphologies of WM1552C cells cultured in 1% ITS medium from passage one (P1) to passage four (P4) over the course of six weeks were monitored by light microscopy. (L-M) Morphologies of MEL-JUSO cells cultured in 1% ITS medium from passage one (P1) to passage two (P2). MEL-JUSO cells failed to proliferate in 1% ITS medium and could not be passaged. Morphologies of live cells were recorded by light microscopy with 40 × objective and 10 × ocular lens. (TIF) [file pone.0215022.s002.tif]

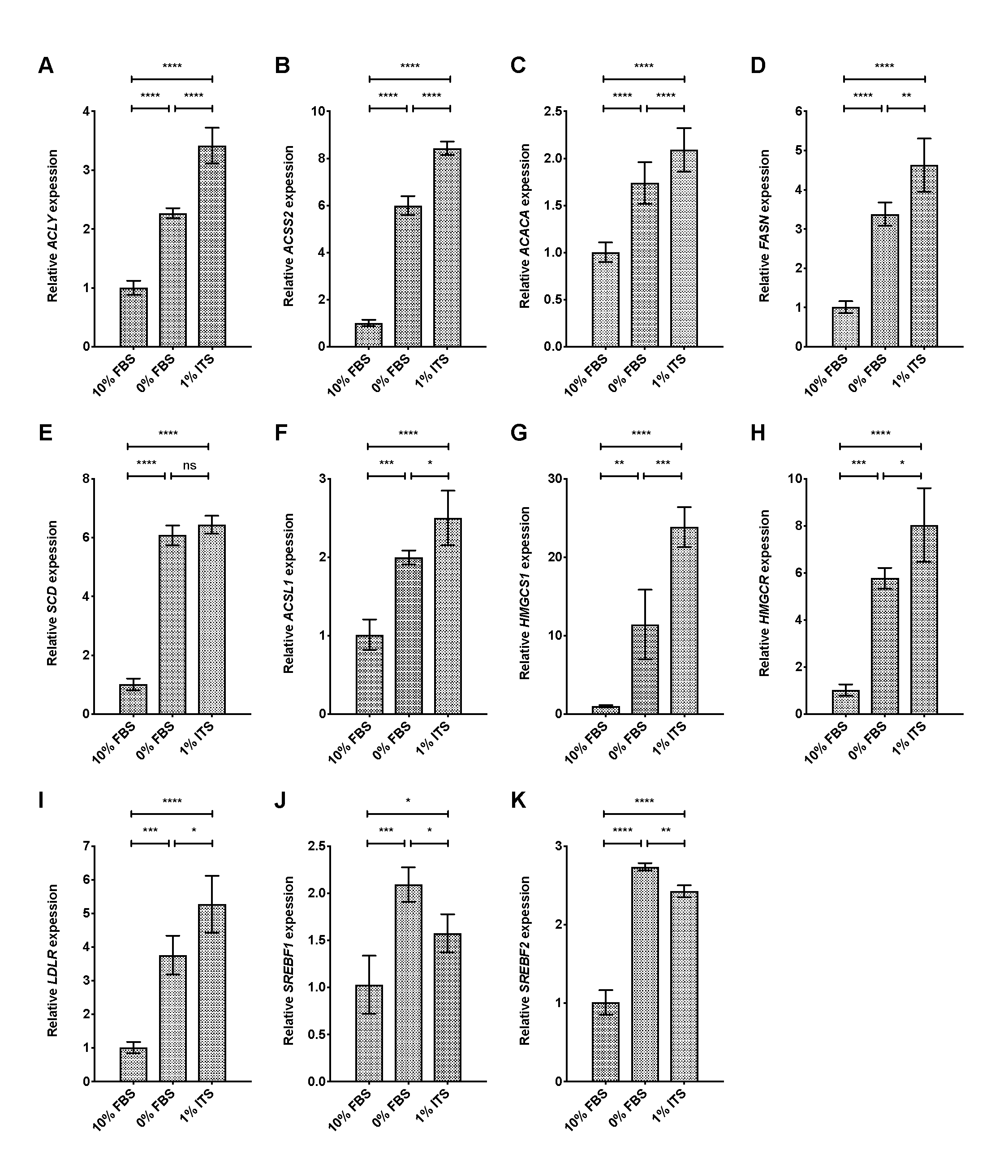

Supplement: S3 Fig — (A-K) The expression level of DNFA and DNCS genes was analyzed by RT-qPCR assay. MEL-JUSO cells were cultured in 10%, 0% FBS or 1% ITS medium for 24 hours. Significant differences between medium conditions are indicated as *P < 0.05, **P < 0.01, ***P < 0.001 and ****P < 0.0001 using one-way ANOVA followed by post hoc Tukey’s multiple comparison tests. ns, not significant. Each data point represents the mean ±SD of results from quadruplicate samples. (TIF) [file pone.0215022.s003.tif]
